# Survivin Modulates Squamous Cell Carcinoma-Derived Stem-Like Cell Proliferation, Viability and Tumor Formation *in Vivo*

**DOI:** 10.3390/ijms17010089

**Published:** 2016-01-12

**Authors:** Roberta Lotti, Elisabetta Palazzo, Tiziana Petrachi, Katiuscia Dallaglio, Annalisa Saltari, Francesca Truzzi, Marika Quadri, Mario Puviani, Antonino Maiorana, Alessandra Marconi, Carlo Pincelli

**Affiliations:** 1 Laboratory of Cutaneous Biology, Department of Surgical, Medical, Dental and Morphological Sciences, University of Modena and Reggio Emilia, via del Pozzo 71, 41124 Modena, Italy; roberta.lotti@unimore.it (R.L.); elisabetta.palazzo@unimore.it (E.P); tiziana.petrachi@virgilio.it (T.P.); katiuscia.dallaglio@yahoo.it (K.D.); annalisa.saltari@unimore.it (A.S.); francesca.truzzi@unimore.it (F.T.); marika.quadri@unimore.it (M.Q.); alessandra.marconi@unimore.it (A.M.); 2 Ospedale Civile di Sassuolo, Via Francesco Ruini 2, 41049 Sassuolo (MO), Italy; mar.puviani@ospedalesassuolo.it; 3 Department of Laboratories and Pathologic Anatomy, University of Modena and Reggio Emilia, Via del Pozzo 71, 41124 Modena, Italy; antonino.maiorana@unimore.it

**Keywords:** squamous cell carcinoma, stem cells, β_1_-integrin, tumor formation, survivin, rapidly adhering cells, skin, tumorigenesis, differentiation

## Abstract

Squamous Cell Carcinoma-derived Stem-like Cells (SCC-SC) originate from alterations in keratinocyte stem cells (KSC) gene expression and sustain tumor development, invasion and recurrence. Since survivin, a KSC marker, is highly expressed in SCC-SC, we evaluate its role in SCC-SC cell growth and SCC models. Survivin silencing by siRNA decreases clonal growth of SCC keratinocytes and viability of total, rapidly adhering (RAD) and non-RAD (NRAD) cells from primary SCC. Similarly, survivin silencing reduces the expression of stem cell markers (OCT4, NOTCH1, CD133, β_1_-integrin), while it increases the level of differentiation markers (K10, involucrin). Moreover, survivin silencing improves the malignant phenotype of SCC 3D-reconstruct, as demonstrated by reduced epidermal thickness, lower Ki-67 positive cell number, and decreased expression of MMP9 and psoriasin. Furthermore, survivin depletion by siRNA in Ras^G12V^-IκBα-derived tumors leads to smaller tumor formation characterized by lower mitotic index and reduced expression of the tumor-associated marker HIF1α, VEGF and CD51. Therefore, our results indicate survivin as a key gene in regulating SCC cancer stem cell formation and cSCC development.

## 1. Introduction

Cutaneous squamous cell carcinoma (cSCC) represents one of the most frequent skin cancers, and together with the basal cell carcinoma (BCC), account for over 1 million new cancers annually in the United States [1]. Surgical resection is curative in 95% of patients with early diagnosis and leads to complete eradication of the tumor. However, this neoplasia may display aggressive histopathologic behavior and undergo relapse and/or perineural or lymphovascular invasion, suggesting that further studies on the mechanisms underlying cSCCs formation are needed.

The most recent models explaining cellular origin and maintenance of tumors indicate a population of stem-like cells, also defined cancer stem cells (CSC), as a “starting point nucleus” for the development of a neoplasia, thus determining its heterogeneity and complexity and affecting tumor development, metastatic behavior and recurrence [2,3,4]. In skin, the oncogenic transformation of the keratinocyte stem cells (KSC), due to genetic or epigenetic alterations [5], gives origin to the SCC-derived Stem-like Cells (SCC-SC), which are responsible for the development of papillomas and cSCC [4]. 

Since the epidermis is a continuously renewing tissue, it is reasonable that any damage occurring in any cells other than stem cells will be rapidly eliminated during the process of epidermal differentiation or apoptosis. Recent studies involving the use of the two-step mouse model of skin carcinogenesis, supports the fact that KSC are targets of both chemical and physical carcinogens and promoters, and determine the formation of papillomas and carcinomas [6]. In particular, cSCC easily develop from the graft of v-ras^Ha^ or v-ras^Ha^–IκBα transformed keratinocytes onto nude mice [7,8,9], and the slow-cycling, quiescent, poorly differentiated (stem cells), but not the rapidly proliferating transit amplifying cells, give rise to more aggressive and invasive tumor [6,10], therefore acting, once transformed, as cancer stem cells. 

The isolation of normal KSC is complicated by the absence of definite markers; however, several molecules, such as β_1_-integrin/CD46, α_6_-integrin/CD71 or survivin [11] have been successfully used to identify KSC and to obtain a population enriched in KSC in culture [12,13,14]. 

In particular, survivin, an IAP protein, is specifically expressed by the high proliferative KSC subpopulation [15] and dually acts on cell cycle regulation and as anti-apoptotic molecule. Survivin functions depend on its intracellular localization [16]: while nuclear survivin is mostly associated with cell division complexes and is essential to mitosis, the mitochondrial/cytoplasmic pool negatively affects caspase activation and is considered as cytoprotective [17]. The high level of survivin is predictive for higher malignancy or poor outcome when analyzed in epithelial tumors [18,19,20,21]. In particular, precancerous and cancerous skin lesions, including cutaneous SCC, presents higher levels of nuclear survivin that is associated with a lower differentiation and a more invasive phenotype [22].

Taking advantage of the KSC isolation method based on β_1_-integrin expression and adhesion to collagen IV-coated plates, primary human cSCC subpopulations with specific properties within the tumor context, have been recently isolated and characterized [22]. These cells have been divided in rapidly adhering (RAD) and non-RAD (NRAD) cells. In particular, RAD cells present stem cell features and work as SCC-SC. They show higher clonogenic ability and, once inoculated in mice, they generate bigger tumors with highly aggressive behavior. 

In this study, we evaluated the role of survivin within primary human cSCC, by analyzing RAD and non-RAD cSCC keratinocytes, as well as cSCC bulk cells. We present evidence that survivin expression increases the malignant phenotype of cSCC, since its functions specifically interfere with RAD cell viability and proliferation as well as with tumor development *in vivo*. 

## 2. Results and Discussion

### 2.1. Survivin Expression Affects cSCC Severity in Vitro

We had previously demonstrated that survivin expression, which identifies KSC *in vitro*, is higher in RAD keratinocytes with respect to NRAD and cSCC bulk cells [22]. This is in agreement with the increased levels of survivin in tumors, in fetal tissues and in stem cells from normal and pathological adult tissues [23,24], and correlates with survivin expression in cSCC lesions [25]. In addition, in keeping with these findings, survivin is involved in chemo and radio-resistance in cancer stem cells [26] and regulates the balance between survival and apoptosis in neural stem cells and glioma CSC [27]. This is functionally relevant to support survivin role within cSCC development and cSCC-SC maintenance.

**Figure 1:**
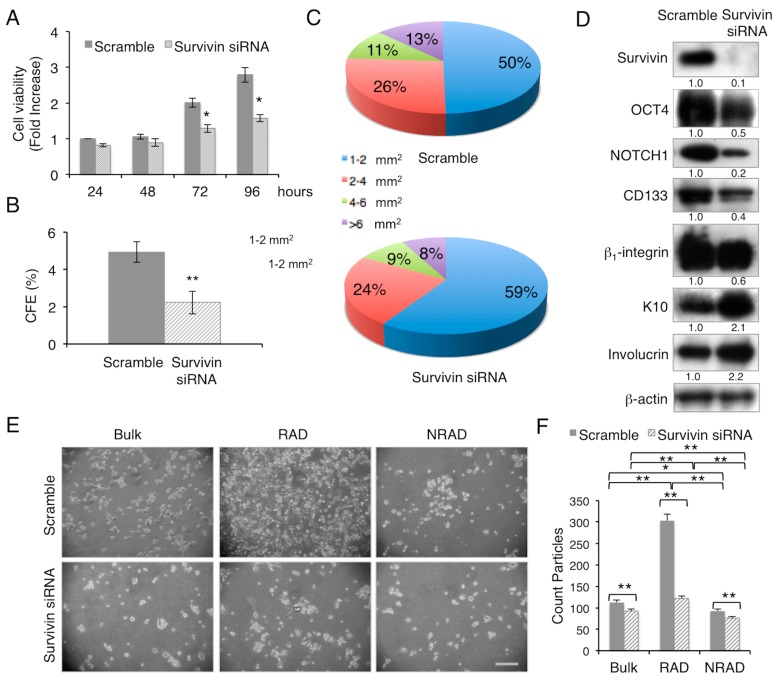
Survivin inhibition decreases cSCC cell proliferation, clonogenic ability and stemness features. (**A**) cSCC cell ability to proliferate *in vitro* after transfection with scramble or survivin siRNA was evaluated by MTT (3-4,5-dimethylyhyazol-2-y1)-2,5-diphenyltetrazolium bromide) assay; (**B**) Clonal growth assessment of cSCC cells after transfection with scramble or survivin siRNA by CFE. CFE was performed in triplicate in three independent experiments and quantified; ** *p* < 0.01; (**C**) Percentage of colonies with respect to size obtained from CFE assay; (**D**) Expression of stem cell and differentiation markers in scramble or survivin siRNA transfected cSCC cells. Cells were analysed 24 h after transfection, and levels of markers were determined by Western blot analysis. β-actin was used as loading control; (**E**) Representative pictures of RAD, NRAD and cSCC bulk cells after transfection with scramble or survivin siRNA, 24 h post-treatment. Scale bar = 200 µm; (**F**) Relative cell density evaluated by ImageJ software analysis (* 0.01 < *p* < 0.05; ** *p* < 0.01).

To evaluate the facets of survivin role in cSCC keratinocytes, we utilized a gene expression silencing approach, by survivin siRNA, to remove the endogenous survivin in cSCC cells from primary tumors. While scramble transfected cells grow exponentially with time, survivin depleted cells display a slow rate of proliferation, as shown by vital cell analysis (Figure 1A), and give rise to a significantly lower amount of colonies with bigger size (Figure 1B,C). In association with the short and long term proliferative potential, survivin expression knocking-down in cSCC primary cells correlates with the decreased expression of stemness-associated markers, such as OCT-4, NOTCH1, CD133 and β_1_-integrin [22,28,29,30,31] (Figure 1D). Interestingly, NOTCH1-survivin axis in the maintenance of human keratinocyte stemness [30] appears to be preserved also in the context of tumor cells. On the other hand, the expression of early epidermal differentiation markers, such as keratin 10 (K10) and involucrin, was increased (Figure 1D). Moreover, by looking closely at the cSCC subpopulation, survivin silencing has the strongest effect on RAD cell density with respect to cSCC bulk and NRAD cells (Figure 1E,F). These results provide evidence of the important role of survivin in the preservation of cSCC-SC pool, breaking down their proliferative potential and, therefore, influencing cSCC tumor aggressiveness.

### 2.2. Survivin Downregulation Decreases SCC Aggressiveness in 3D-Models

To better define how survivin expression correlates with cSCC morphology and differentiation, we prepared 3D skin reconstructs from SCC13-derived RAD and NRAD cells after transfection with survivin siRNA. The efficiency of survivin silencing as compared to control was evaluated by immunohistochemical staining and survivin positive cell count (data not shown). 

**Figure 2:**
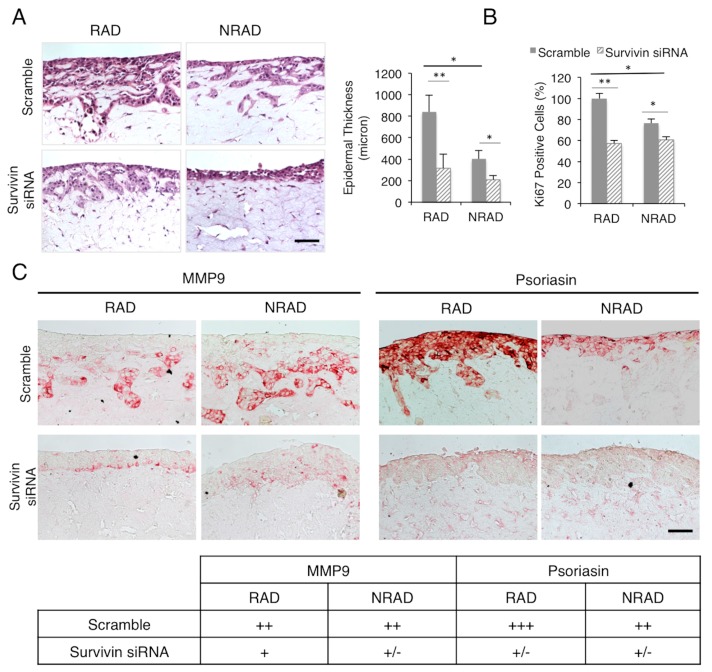
Survivin depleted RAD and NRAD-derived skin reconstructs. (**A**) Left panel: Hematoxilin and Eosin staining of survivin siRNA-treated RAD and NRAD SCC13 derived skin reconstructs. Right panel: Bar graph representing epidermal thickness (micron) of survivin siRNA-treated RAD and NRAD SCC13 derived skin reconstructs. Scale bar = 120 µm; (**B**) Bar graph representing the number of Ki-67 positive cells; (**C**) MMP9 and psoriasin expression evaluated by immunohistochemistry in siRNA-treated RAD and NRAD SCC13 derived skin reconstructs, with the table indicating the relative scores: - not expressed; + poorly expressed; ++ moderately expressed; +++ well expressed. The images are representative of three independent experiments. (* 0.01 < *p* < 0.05; ** *p* < 0.01). Scale bar = 120 µm.

It has been shown previously that skin equivalents generated with cSCC cell lines display extensive dermal invasion and altered attachment of the epidermal keratinocytes to the dermis [32]. These 3D reconstructs were characterized by epidermal hyperproliferation, higher expression of proliferation-related markers and misregulated differentiation. In our model, we were able to recapitulate the morphological features of cSCC. At the histological level, both RAD and NRAD-derived skin equivalents showed squamous keratinocytes, epidermal hyperplasia and dermal invasiveness. Survivin-depleted cSCC cells gave origin to a thinner epidermis, with significant reduction of epidermal thickness in RAD-derived skin equivalents, and decreased dermal-invading areas (Figure 2A). 

Consistently, survivin silencing decreased the number of Ki-67 positive cells in both RAD and NRAD SCC reconstructs (Figure 2B), thus demonstrating survivin ability to modulate the proliferative capacity of SCC13 keratinocytes in the 3D skin model *in vitro*. Most importantly, the proliferative brake was more efficient in RAD with respect to NRAD SCC reconstructs, whose derived tumors have been previously shown to have a lower number of Ki-67 positive cells and a reduced mitotic index, as compared to RAD tumors [22]. 

The release of metalloproteinase-9 (MMP-9), which correlates with invasion and metastasis [33,34], resulted significantly reduced in survivin-silenced SCC13 skin equivalent (Figure 2C). These findings are in agreement with the demonstration that, in the presence of survivin overexpression, cSCC are more aggressive and display higher metastatic potential, thus correlating with a poor prognosis [25]. On the other hand, in the absence of survivin, the malignant phenotype of cSCC epidermal reconstructs was significantly ameliorated, as demonstrated by the strongly decrease of S100A7 (psoriasin) expression (Figure 2C), which is a recognized marker of poor prognosis in epithelial cancers and is significantly upregulated in human epithelial skin tumors [35,36]. 

Therefore, these data demonstrate that survivin affects SCC cancer cell stemness property, by regulating the proliferative potential and aggressiveness of SCC cells. 

### 2.3. Survivin Expression Is Crucial in Tumor Aggressiveness

Human skin cancers, such as BCC and SCC, mostly arise from KSC coming from interfollicular locations in human skin [37,38,39]. About 20%–25% of sporadic cSCC lesions have activating mutation in RAS [40] and the expression of the Ha-RasG12V allele in normal human keratinocytes produce a set of biochemical and morphological modifications, which lead to cell cycle block at the G1 phase and to the accumulation of senescent cells [41]. This block could be bypassed by using CDK4 (cyclin-dependent kinase 4) overexpression or the inhibition of NFkB activation through IκBα [9,41]. The coexpression of Ras^G12V^ and IκBα (Ras^G12V^-IκBαM) in human keratinocytes used to regenerate skin in immuno-deficient mice promote the formation of rapidly growing tumors resembling SCC [9].

Therefore, in order to analyze the role of survivin in human cSCC formation and progression *in vivo*, we used a model where human primary neonatal keratinocytes were infected with retroviral vectors expressing the oncogenic Ras (Ha-Ras^G12V^) and the inhibitor of NFκB (IκBαM), and subsequently transfected with survivin siRNA before injection on the flank skin of CB17-SCID mice. This model allows a direct evaluation of the malignant transformation of human keratinocytes, therefore limiting the differences between human and mouse skin in the context of tumor development [37].

**Figure 3:**
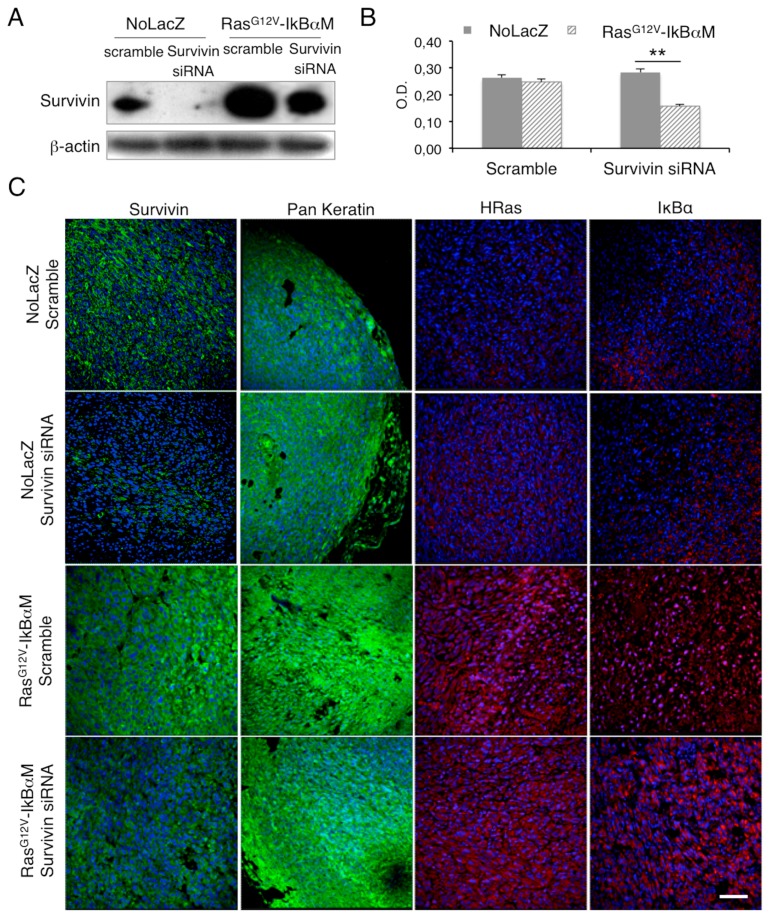
Survivin silencing decreases cell viability in Ras^G12V^-IκBαM-transduced keratinocytes. (**A**) Expression of survivin in scramble or survivin siRNA transfected NoLacZ or Ras^G12V^-IκBαM-transduced keratinocytes. Cells were analysed 48 h after transfection, and levels of markers were determined by Western blot analysis. β-actin was used as loading control; (**B**) Survivin siRNA transfected NoLacZ or Ras^G12V^-IκBαM-transduced keratinocytes ability to proliferate *in vitro* evaluated by MTT assay 72 h after transfection; (**C**) Survivin, pan-keratin, H-Ras and IκBα staining in survivin siRNA transfected NoLacZ or Ras^G12V^-IκBαM tumors evaluated by immunofluorescence. (** = *p* < 0.01). Scale bar = 200 µm.

After checking survivin depletion by Western Blot analysis, we found that the expression of Ha-Ras^G12V^-IκBαM in keratinocytes promoted survivin upregulation (Figure 3A), which is in line with the induction of the oncogenic phenotype in keratinocytes and the higher level of survivin in cancer cells [42]. This is also in agreement with Ras-dependent upregulation of survivin in HaCaT cells [43,44] and normal K-Ras promoted survivin degradation [45]. 

According to survivin role in promoting RAD/cSCC-SC maintenance, survivin depletion in Ras^G12V^-IκBαM-transduced keratinocytes produced a statistically significant reduction of cell proliferation (Figure 3B). These cells were successfully used for tumorigenesis experiments *in vivo*. Tumors derived from survivin-silenced Ras^G12V^-IκBαM-transduced keratinocytes and control cells (referred as NoLacZ) were evaluated for survivin, keratins, H-Ras and IκBαM expression to confirm the epithelial origin and the efficiency of infection (Figure 3C). While there is no difference in size between tumors generated by survivin or scramble siRNA treated control cells, Ras^G12V^-IκBαM keratinocytes give rise to a substantially smaller tumor in the absence of survivin silencing (Table 1). Moreover, in the presence of survivin siRNA, all cases (3/3) of injected Ras^G12V^-IκBαM keratinocytes do not create tumors at four weeks (Table 1). 

**Table 1:** Evaluation of *in vivo* tumor formation after sub-cutaneous injection of human keratinocytes infected with retroviral vector expressing Ha-Ras^G12V^ and IκBαM in presence or in absence of endogenous survivin.

Condition	Tumour at 4 Weeks	Tumour at 6 Weeks	Tumour Size (cm^3^ ± SD)
NoLacZ Scramble	− (3/3)	+ (3/3)	0.49 ± 0.17
NoLacZ Survivin siRNA	− (3/3)	+ (3/3)	0.39 ± 0.14
Ras^G12V^-IκBαM Scramble	+ (3/3)	+ (3/3)	0.89 ± 0.33
Ras^G12V^-IκBαM Survivin siRNA	− (3/3)	+ (3/3)	1.01 ± 0.38

**Figure 4:**
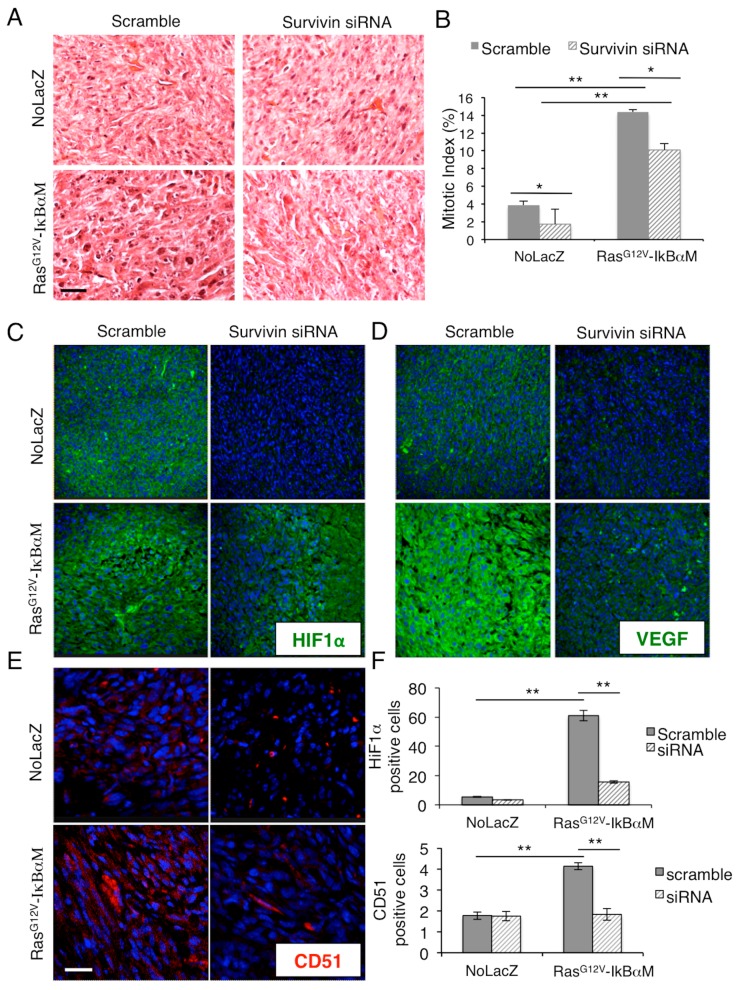
Survivin silencing decreases the malignant phenotype of Ras^G12V^-ΙκΒαΜ-derived tumors. (**A**) Hematoxilin and Eosin staining of survivin siRNA-treated Ras^G12V^-ΙκΒαΜ-derived tumors; (**B**) Mitotic Index representing the number of cells undergoing mitosis over total cells. Scale bars = 200 µm; (**C**) HIF1α staining in survivin siRNA transfected NoLacZ or Ras^G12V^-ΙκΒαΜ tumors evaluated by immunofluorescence; (**D**) VEGF staining in survivin siRNA transfected NoLacZ or Ras^G12V^-ΙκΒαΜ tumors evaluated by immunofluorescence; (**E**) CD51 staining in survivin siRNA transfected NoLacZ or Ras^G12V^-ΙκΒαΜ tumors evaluated by immunofluoresce; (**F**) HIF1α (**upper** panel) and CD51 (**lower** panel) positive cell count assessed by ImageJ software (* 0.01 < *p* < 0.05; ** *p* < 0.01). Scale bars = 200 µm.

Moreover, Ras^G12V^-ΙκΒαΜ/scramble siRNA-tumors displayed less alterations at the nuclear level with respect to Ras^G12V^-ΙκΒαΜ/survivin siRNA-tumors (Figure 4A), with bigger nuclei and prominent nucleoli, which correlates with survivin implications in chromosomal stability and segregation [46], as well as a higher mitotic index (Figure 4B). Moreover, the analysis of the cancer associated markers HIF-1α, VEGF and CD51 (α_v_-integrin) [47,48,49] strongly support the implication of survivin overexpression in favor of a poor tumor prognosis. In particular, the upregulation of HIF-1α and VEGF promoted by Ras^G12V^-ΙκΒαΜ was inhibited by survivin depletion (Figure 4C,D,F upper panel). Similarly, the number of CD51 positive cells that was increased by Ras^G12V^-ΙκΒαΜ expression, was restored to NoLacZ levels in Ras^G12V^-ΙκΒαΜ/survivin siRNA tumors (Figure 4E,F lower panel). These results further indicate the critical role of survivin within skin tumorigenesis and the importance of targeting survivin in SCC-SC in order to get a significative improvement of the cutaneous neoplasia.

## 3. Materials and Methods

### 3.1. Isolation of Primary Keratinocytes from cSCC Tissues

Primary keratinocytes from human cSCC were isolated as previously described [22]. Briefly, ten tumor samples (as biological replicates) from human cSCC patients were surgically removed and immediately stored in a sterile test tube containing medium and antibiotics. All tumor samples were collected with written informed consent of patients, according to the Declaration of Helsinki after approval of the Modena Medical Ethical Committee. Tumor tissues were washed with PBS without calcium and magnesium, cut into small fragments and digested in DMEM (Dulbecco’s Modified Eagle Medium) containing 200 U/mL type I collagenase, 200 U/mL dispase and 70 U/mL DNase shaking for 2 h at 37 °C. The digested top tissue mixture was then filtered and centrifuged to collect the cells. Total cells were then seeded onto 3T3 feeder layers as previously described [50] and primary and secondary cell cultures were obtained. 

For RAD and NRAD cells isolation, collagen IV coated plates were prepared by seeding a human placenta-derived collagen IV solution (100 μg/mL, Sigma-Aldrich, St. Louis, MO, USA). Total cells from cSCC cultures, either at passage 0 or 1, were seeded on collagen IV pre-coated dishes for 5 min. Cells adhering within 5 min represent RAD cSCC keratinocytes; in order to analyze RAD and NRAD cellular morphology, after cell separation, NRAD and bulk cells were collected and seeded on a dish of the same size as the one used for the separation; pictures have been taken within 10 min from the separation process. For *in vitro* proliferation/viability, clonogenic assays or 3D skin reconstruct after transfection with scramble or survivin siRNA (Dharmacon Inc, Lafayette, CO, USA) cells were maintained in culture with serum-free keratinocyte growth medium (KGM) until they reached the desired confluence. 

### 3.2. Isolation of Primary Keratinocytes from Healthy Skin and SCC Cell Line Culture

Normal human keratinocytes were isolated from healthy skin biopsies obtained from waste materials from Surgical Room. Patient consent for experiments was not required because Italian laws consider human tissue left over from surgery as discarded material. Isolated cells were cultured as described by Pincelli and colleagues [15]. Cells were maintained in culture with KGM until confluence and utilized for *in vivo* tumorigenesis assay. SCC13 cell line were purchased from ATCC (Teddington, UK) and maintained in culture with KGM. 

### 3.3. siRNA Transfection of Keratinocytes

cSCC cells (RAD, NRAD or bulk cells), human keratinocytes transduced with Ras^G12V^-ΙκΒαΜ or control vector (NoLacZ) or SCC13 were plated in antibiotic-free KGM medium in order to get sub confluent conditions 24 h post-seeding. Cells were transfected twice with 25 nM scrambled or survivin siRNA (ON-TARGET plus SMARTpool code L003459, Dharmacon Inc., Lafayette, CO, USA), combined with Lipofectamin 2000 and Opti-MEM (Thermo Fisher, Waltham, MA, USA), as the datasheet suggests. Cells were evaluated at different time points or further seeded according to the specific experiment requirements.

### 3.4. Detection of Cell Viability by MTT

cSCC bulk cells (5000/well) or human keratinocytes transduced with Ras^G12V^-ΙκΒαΜ or control vector (NoLacZ) were seeded in a 96-wells plate and transfected with scramble or survivin siRNA 24 h post plating. MTT (3-(4,5-dimethylthiazol-2-yl)-2,5-diphenyltetrazolium bromide) (Sigma-Aldrich, St. Louis, MO, USA) assay was performed at 24 h, 48 h, 72 h and 96 h post transfection or 72 h, according to the specific experiment requirements. The results are expressed as fold increase respect to scramble treated cells at 24 h or as Optical Density (OD) plotted against time. Results are calculated as the mean SD of three different experiments. 

### 3.5. Colony Forming Efficiency (CFE)

cSCC bulk cells were transfected with scramble or survivin siRNA and subcultured on a feeder layer composed of mytomicin C (Sigma-Aldrich, St. Louis, MO, USA)-treated 3T3 cells at a density of 100 cells per dish 24 h post-transfection. Fourteen days later, dishes were fixed with 10% buffered formalin and stained with crystal violet. Colonies were manually scored. The results were expressed as percentages of the number of cells plated in each dish or number of colonies for each size and represented as the mean ± SD of three independent experiments. 

### 3.6. Western Blotting (WB)

Total proteins from siRNA-transfected cSCC bulk cells or siRNA-transfected Ras^G12V^-ΙκΒαΜ or control vector (NoLacZ) were extracted with RIPA (Radioimmunoprecipitation assay) lysis buffer containing protease inhibitors. Equal amounts of protein from each sample were run through a 6%–18% SDS–PAGE gel and transferred onto a nitrocellulose membrane. Briefly, membranes were incubated overnight at 4 °C with the following primary antibodies: rabbit polyclonal anti-human survivin (1:1000; Novus Biologicals, Littleton, CO, USA), or rabbit polyclonal anti-human NOTCH1 (1:500; Abcam, Cambridge, UK), or rabbit anti-human OCT4 (1:1000; Novus Biologicals, Littleton, CO, USA), mouse anti-human CD133 (1:200; Miltenyi Biotec Inc., Auburn, CA, USA), mouse anti-human β_1_-integrin (1:500; Santa Cruz Biotechnology Inc., Santa Cruz, CA, USA), or mouse monoclonal anti-human involucrin (1:1500; Sigma-Aldrich), or rabbit polyclonal anti-human Keratin 10 (1:5000; Epitomics Burlingame, CA, USA) or mouse monoclonal anti-human β-actin (1:5000; Sigma-Aldrich). After 3 washes with a PBS/tween solution, membranes were then incubated with secondary antibodies: goat anti-mouse or goat anti-rabbit (1:3000; Bio-Rad Laboratories, Hercules, CA, USA) for 45 min at room temperature. Bands were then visualized with chemiluminescence detection system (Amersham Biosciences UK Limited, Little Chalfont Buckinghamshire, UK). The band intensity was quantitatively determined using ImageJ software (Wayne Rasband, National Institute of Mental Health, Bethesda, MD, USA), and protein levels’ intensity was normalized to β-actin expression.

### 3.7. H and E Staining and Mitotic Index Calculation

cSCC skin reconstruction or tumors were removed from animals, placed in 4% buffered formalin for no more than 24 h, and then embedded in paraffin. Serial tissue sections (4 μm thick) were deparaffinized, hydrated in xylene and graded alcohol solutions. For H and E stains, Mayer hematoxylin and Eosin Y Alcoholic were used. Staining times were 5 min for hematoxylin and 1 min for eosin. Slides were covered with coverslips with the addition of aqueous mount. The mitotic index was calculated and expressed as the ratio between the number of cells in mitosis and the total number of cells on H and E sections.

### 3.8. Immunohistochemistry

cSCC skin reconstruct were fixed with formalin for 2 h at room temperature, dehydrated and embedded in paraffin. The staining was performed using the UltraVision LP Detection System AP Polymer & Fast Red Chromogen assay (Thermo Fisher Scientific, Waltham, MA, USA), according to the manufacturer’s instructions. Briefly, slides were treated with Ultra V Block and samples were incubated with anti-Ki-67 (1:200; Epitomics, Burlingame, CA, USA), or anti-keratin (ready to use; Cell Marque), or anti-MMP9 (1:100; Abcam, Cambridge, UK) or anti-psoriasin (1:50, Abcam, Cambridge, UK) for 1 h at room temperature. 

After washes in PBS, Primary Antibody Enhancer (Thermo Fisher Scientific, Waltham, MA, USA) was added for 20 min at room temperature, followed by incubation with AP Polymer anti-mouse/rabbit IgG for 30 min at room temperature. Slides were stained with Fast Red using Naphthol Phosphate as substrate. Samples were analyzed under a conventional optical microscope (Zeiss Axioskope 40, Carl Zeiss, Jena, Germany).

### 3.9. Immunofluorescence

Four micrometer skin sections from formalin fixed-paraffin tumors were rehydrated in PBS buffer and permeabilized by incubation for 10 min with 0.5% Triton X-100. Then, slides were incubated for 15 min with 0.5% bovine serum albumin and 5% goat serum, and for 60 min at 37 °C with the rabbit polyclonal anti-human Survivin antibody (1:50, Novus, Bloomington, MN, USA) or HIF1α (1:500, Novus, Bloomington, MN, USA), VEGF (1:50, Thermo Fisher Scientific, Waltham, MA, USA), CD51 (1:50, Abcam, Cambridge, UK). After four washes in PBS, samples were incubated for 60 min with the anti-rabbit secondary antibody, Alexa Fluor 546 (1:100, Thermo Fisher Scientific, Waltham, MA, USA). Fluorescent specimens were analyzed by confocal scanning laser microscope (Leica TCS SP2) and positive cell counts were performed by ImageJ software analysis.

### 3.10. Skin Reconstruct

Skin reconstruct from RAD, NRAD and bulk SCC13 cells transfected with scramble or survivin siRNA were obtained as previously described [51]. For dermal reconstructs, 0.5 mL of a cell free collagen solution (1.35 mg/mL rat tail collagen type I in DMEM with 10% FCS and 1% Pen/Strep) was added to tissue culture inserts (Transwell, Costar, Cambridge, MA, USA) in 12-well plates. This pre-coated acellular layer was overlaid with 1 mL of fibroblasts mixed with collagen type I solution (15 × 10^4^/mL). After 4 days of incubation at 37 °C, dermal reconstructs were rinsed and equilibrated with keratinocyte medium for 1 h at 37 °C. SCC13 cells (25 × 10^4^ cells) were seeded onto the concave of the dermal reconstruct, incubated for 1 h at 37 °C to allow attachment of the cells and then submerged in keratinocyte medium 40 for 4 days. Finally, skin reconstructs were exposed to the air and EGF free keratinocyte medium was changed every two days. After 12 days, skin reconstructs were fixed with formalin for 2 h at room temperature, dehydrated and embedded in paraffin.

### 3.11. In Vivo Tumorigenesis

Freshly isolated human primary keratinocytes were infected twice with retroviral vectors expressing Ras^G12V^ and IκBα or control vector (NoLacZ) (kindly provided by Prof. P.A. Khavari, Stanford University, Stanford, CA, USA). 24 h from the last infection, cells were further transfected with scramble or survivin siRNA, as previously indicated. 24 h post transfection, 10^6^ cells were mixed with 50 mL of Matrigel (BD Bioscience, San Jose, CA, USA) and injected in the dorsal fascia of recipient CB17 SCID mice. 

Tumors were harvested when they reached 1 cm diameter size for histological analysis, mitotic index measure and immunostaining. Tumor volume was calculated by the formula (longest diameter) × (shortest diameter)^2^/2.

### 3.12. Statistical Analysis

The Student’s *t*-test was used to compare the average intensities of WB bands, average viabilities and average cell counts. One (*) or two (**) asterisks indicate a significant difference, 0.01 < *p* < 0.05 and *p* < 0.01, respectively.

## 4. Conclusions

Overall, our data shed light on the role of survivin in the context of squamous cell carcinoma-derived stem like cells. We found that survivin silencing decreased cell viability and proliferation of RAD cells, which have been previously identified as cancer stem cells in cutaneous SCC [22,25]. Unlike other KSC markers, survivin plays a critical role in both cell cycle regulation and apoptosis [52], which are dysregulated in cancer, thus being the best candidate to drive development of SCC in the skin. This suggests that survivin could be a key gene of skin cancer stem cells. Our study suggests that blocking survivin could be successfully used to treat cSCC through targeting cancer stem cells, thus inhibiting development, metastasis and recurrence of tumors [53].
